# Strengthening comprehensive tuberculosis care in Angola: a scoping review of strategies, challenges, and potentialities in a high-burden setting

**DOI:** 10.3389/phrs.2026.1609466

**Published:** 2026-08-27

**Authors:** Valter Chicalo António Caripa, Rubia Laine de Paula Andrade-Gonçalves, Rafaele Oliveira Bonfim, Mariana Gaspar Botelho Funari de Faria, Yan Mathias Alves, Reginaldo Bazon Vaz Tavares, Titilade Kehinde Ayandeyi Teibo, Willie Otavio Bueno Bernardi, Rander Junior Rosa, Ariela Fehr Tártaro, Natacha Martins Ribeiro, André Luiz Teixeira Vinci, Nathalia Zini, Ricardo Alexandre Arcêncio

**Affiliations:** 1 Department of Maternal-Infant and Public Health Nursing, Ribeirão Preto College of Nursing, University of São Paulo, Ribeirão Preto, Brazil; 2 Polytechnic Institute of Saurimo, Lueji A’Nkonde University, Saurimo, Angola; 3 Brazilian Tuberculosis Research Network (REDE-TB), Rio de Janeiro, Brazil

**Keywords:** Angola, comprehensive healthcare, delivery of healthcare, public health surveillance, tuberculosis

## Abstract

**Objectives:**

To identify surveillance and care actions implemented for tuberculosis control in the country, as well as to analyze factors that hinder or facilitate their implementation.

**Methods:**

This scoping review followed the methodology recommended by the Joanna Briggs Institute (JBI) Manual for Scoping Reviews and the PRISMA Extension for Scoping Reviews (PRISMA-ScR). The protocol was registered in the Open Science Framework (OSF). The review question was formulated based on the PCC acronym, and searches were conducted in PubMed, Embase, LILACS, Web of Science, Scopus, and Google Scholar.

**Results:**

Of the 564 records identified, 13 studies were included, encompassing 24,652 participants, including adults and children. Regarding prevention, BCG vaccination achieved approximately 90% coverage among children under 5 years of age. Diagnostic approaches included clinical evaluation, chest radiography, and laboratory tests (AFB smear microscopy/GeneXpert), while major challenges included shortages of supplies (reported in 100% of the studies) and underreporting.

**Conclusion:**

Updating guidelines, strengthening professional training, and expanding diagnostic and treatment services are priority measures to consolidate tuberculosis control in the country.

## Introduction

It is estimated that approximately one-quarter of the global population is infected with *Mycobacterium tuberculosis* (MTB), with a risk of progressing to active tuberculosis (TB), thereby sustaining disease transmission and challenging global efforts to end TB as a public health problem [1, 2]. Among the most affected continents, Africa remains a major epicenter of the TB epidemic, accounting for approximately 25% of all newly diagnosed TB cases worldwide, equivalent to 2.5 million cases in 2022. In the same year, the continent accounted for 33% of global TB-related deaths, corresponding to approximately 424,000 deaths [3].

In 2023, Angola reported an estimated TB incidence rate of 325 cases per 100,000 population, with the disease predominantly affecting younger age groups, particularly individuals aged 15–44 years [4]. Several studies have highlighted the persistent and concerning TB burden in the country [5, 6, 7]. However, the available evidence presents important limitations at three levels: (1) empirical gap: the absence of a comprehensive synthesis of strategies, challenges, and potentialities across the TB care continuum in Angola; (2) system-level gap: the lack of integrated analysis linking surveillance, healthcare delivery, and outcomes; and (3) contextual gap: limited evidence from fragile health systems in high TB burden-settings, where structural constraints, social determinants, and health system vulnerabilities shape TB control efforts.

From a health systems perspective [8], this situation reflects not only the magnitude of the TB burden in Angola, but also the limited capacity to prevent disease, ensure early detection, provide timely diagnosis, maintain continuity of care, and deliver effective treatment support and follow-up. These challenges are closely related to structural and operational constraints, including insufficient financing, limited management capacity, weaknesses in surveillance systems, and shortages of essential supplies and human resources. TB outcomes are shaped by the interaction between the pathogen, individual vulnerability, and social conditions, all of which are influenced by how health systems organize, deliver, and sustain care across the continuum over time [9].

Tuberculosis care requires comprehensive approaches that address the multiple stages of the care continuum, including diagnosis, treatment, rehabilitation, and long-term follow-up. These approaches depend on coordinated actions among healthcare providers and services to ensure timely access, continuity of care, and improved treatment outcomes. In this context, Primary Health Care (PHC) plays a central role in coordinating TB-related actions across the health system, linking prevention, diagnosis, treatment, and follow-up services with broader social support networks. This integrated approach is essential to address the social determinants that influence TB vulnerability, access to care, and treatment outcomes [10, 11].

Therefore, understanding comprehensive TB care is essential to identify health system gaps, characterize implementation challenges, and recognize opportunities to strengthen the response to TB, particularly in alignment with the Sustainable Development Goals (SDGs), the 2030 Agenda, and the World Health Organization (WHO) End TB Strategy [12]. This strategy aims to reduce TB deaths by 90% and new TB cases by 80% by 2030 [13], supported by three key pillars: integrated, patient-centred care and prevention; bold policies and supportive systems; and intensified research and innovation [9]. Achieving these targets requires effective coordination and sustained performance of health systems across the TB care continuum, particularly in high-burden settings where structural and operational barriers may compromise access to prevention, diagnosis, treatment, and follow-up.

A literature search was conducted in review registries (PROSPERO and OSF Registries) and PubMed, and no ongoing or published reviews specifically addressing comprehensive TB care in Angola were identified. Therefore, this scoping review aimed to identify the actions developed, challenges, and opportunities for strengthening comprehensive TB care for people with TB in Angola. By synthesizing available evidence across the continuum of care, this review seeks to provide insights into health system gaps and potential strategies to support the development of policies and interventions for TB control in the country.

## Methods

This study followed the methodological guidance provided by the Joanna Briggs Institute (JBI) Manual for Scoping Reviews [14] and the recommendations of the Preferred Reporting Items for Systematic Reviews and Meta-Analyses extension for Scoping Reviews (PRISMA-ScR) [15]. The review protocol was registered in the Open Science Framework (OSF) Registries with the DOI: 10.17605/OSF.IO/G5X9C.

### Review question

The research question —“What is the scientific evidence on strategies, challenges, and opportunities for strengthening comprehensive tuberculosis care in Angola?”—was formulated based on the PCC acronym, where P (Population) refers to the general population; C (Concept) to surveillance and care strategies for tuberculosis control; and C (Context) to the national setting of Angola.

### Eligibility criteria

Original research articles addressing comprehensive TB care strategies and actions in the Angolan context were included, regardless of methodological approach (quantitative, qualitative, or mixed methods) or study design. For this review, comprehensive TB care was defined as a set of coordinated actions across the continuum of care, including prevention, case detection, diagnosis, treatment initiation and monitoring, adherence support, and follow-up. Tuberculosis surveillance actions were considered those related to case identification, notification, monitoring, data management, and evaluation of TB control activities.

The study population included individuals of all ages with suspected or confirmed TB disease and/or TB infection. Studies that analyzed the Angolan context in combination with other countries were excluded. No restrictions regarding publication period were applied to capture the full extent of the available evidence, as no previous reviews addressing comprehensive TB care in Angola were identified. Studies published in Portuguese, English, or Spanish were considered eligible.

### Search strategy

Based on the keywords defined by the PCC framework, controlled vocabulary related to these terms was identified using the Health Sciences Descriptors (DeCS), along with corresponding synonyms in Portuguese, English, and Spanish. For English descriptors, the Medical Subject Headings (MeSH) were also consulted. In addition, preliminary searches were conducted in the databases to identify relevant free-text terms and refine the search strategy, ensuring adequate sensitivity and specificity. The searches were conducted in May 2025 by a librarian trained in systematic literature searching (Supplementary Material Table 1).

### Study selection/source of evidence

All retrieved records were exported to the Rayyan CRI application [16], where duplicate records were initially removed. Before the screening process, reviewers were aligned on the review question and the predefined inclusion and exclusion criteria to ensure consistency in study selection Subsequently, titles and abstracts were independently screened by two reviewers. Disagreements regarding study inclusion were resolved through discussion and consensus, and when necessary, by consultation with a third reviewer for final decision. The full texts of potentially eligible studies were then assessed for relevance and eligibility. The entire search and selection process was documented using a flow diagram in accordance with the Preferred Reporting Items for Systematic Reviews and Meta-Analyses guidelines [17].

### Data extraction

Data extraction was performed independently by three authors and subsequently verified by a fourth reviewer. A structured data extraction form was used, including the following variables: authors, language, year of publication, study location, type of publication, journal, objectives, methodological design, study population and sample, data analysis methods, and main findings. The extracted data included TB surveillance and comprehensive care actions implemented in Angola, as well as factors that hindered or facilitated the implementation of these actions. The data extraction form was tested on a subset of studies and refined as necessary.

### Data analysis and presentation

A thematic synthesis was conducted according to Bardin’s content analysis method, using the findings from the 13 studies included in the review. The analysis comprised the following steps: familiarization with the study findings, identification of meaning units, coding of relevant findings, grouping according to semantic and interpretative similarities, and development of thematic categories [18].

The artificial intelligence tool Claude was used as a supplementary resource to support reflection on and refinement of the interpretations and thematic categories developed by the researchers.

## Results

**FIGURE 1:**
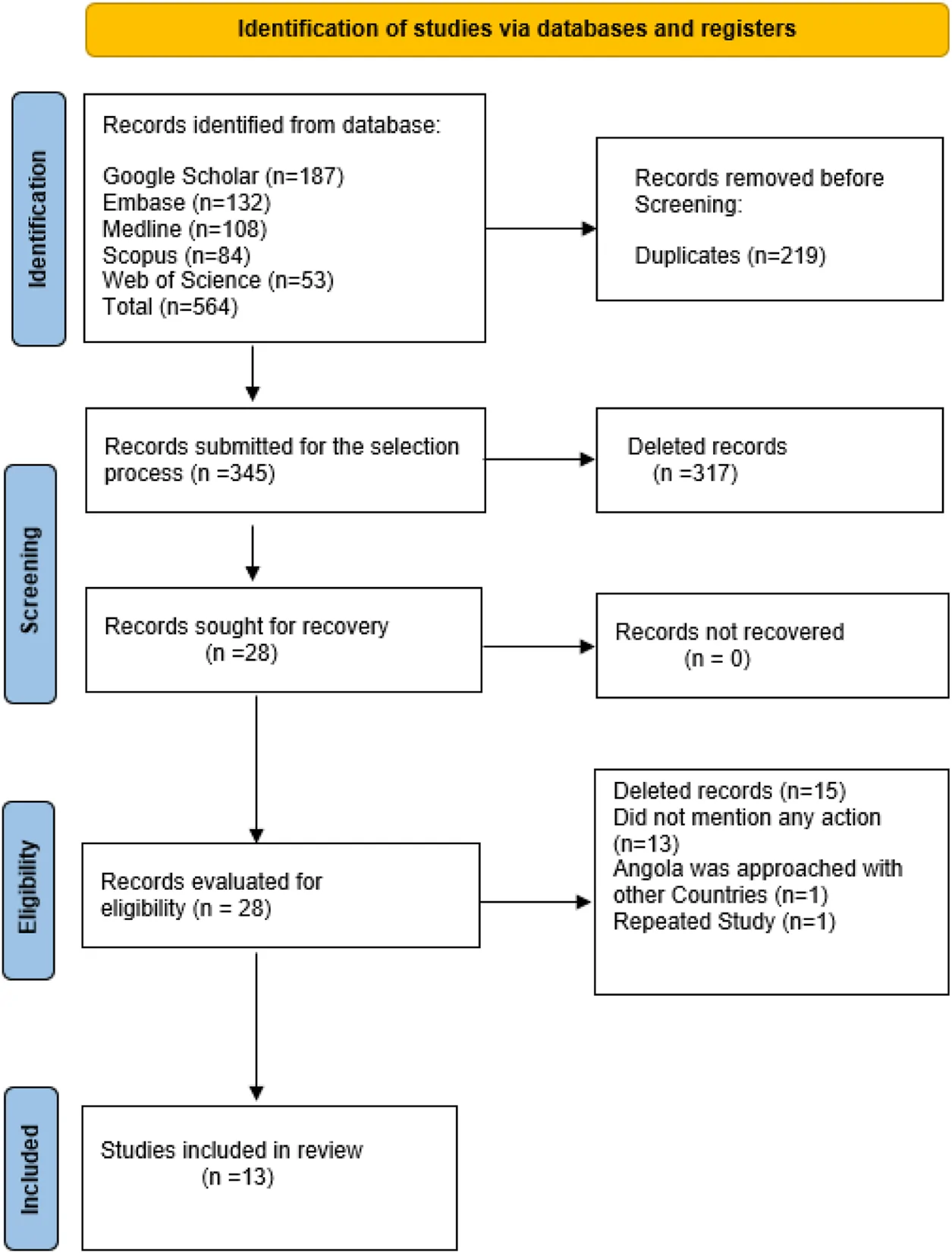
PRISM-ScR diagram flow illustrating the study selection process for the scoping review of TB control measures in Angola, from identification and screening to eligibility assessment and inclusion of studies (Angola, 2025).

A total of 564 publications were identified. Of these, 219 were excluded due to duplication, 317 were excluded after title and abstract screening, and 15 were excluded following full-text assessment. Consequently, 13 studies were included in the review (Figure 1).

The total number of participants across the included studies was 24,652, comprising adults and children under 5 years of age. The study population included index TB cases, people with TB/HIV co-infection, and health professionals. The included studies comprised descriptive designs (39%; n = 3), cross-sectional studies (26%; n = 2), retrospective studies (26%; n = 2), qualitative studies (26%; n = 2), and other designs (52%; n = 4) (Tables 1 and 2).

**TABLE 1:** Characteristics of studies included in the scoping review on comprehensive TB care in Angola (Angola, 2025).

No.	Author/Year of publication	Journal	Objective
1	Martínez-Camprecios et al., 2024 [21]	Bulletin of the world health organization	To evaluate the results of a contact-tracing program aimed at increasing TB diagnosis in Cubal, Angola, and providing preventive treatment to high-risk groups
2	Faust et al., 2020 [22]	Health science reports	To analyze how countries with a high TB burden are implementing policies, guidelines, and tools for the control of latent TB infection (LTBI), assessing current practices, progress, and the main obstacles faced in adopting strategies to diagnose and treat LTBI.
3	Oliveira; Martinez; Rocha, 2014 [19]	Journal of public health	To analyze vaccination coverage and the factors associated with completion of the vaccination schedule among children under 5 years of age
4	Umar; Kabamba, 2016 [20]	Journal of family medicine and primary care	To present a case of double BCG vaccination in a child living in the city of Calai, Cuando Cubango province, Angola
5	Doveren, 2001 [21]	International journal of tuberculosis and lung disease	Describe the experience of implementing DOTS for TB, adapted to the challenging circumstances of a city partially populated by displaced persons
6	Cameia, 2020 [22]	Not applicable	To identify the challenges related to adherence to TB treatment among people living with HIV at a referral center in huambo, Angola, and to identify the strategies used by healthcare professionals to address these challenges in huambo, Angola
7	Sebastião et al., 2022 [23]	American journal of tropical medicine and hygiene	To analyze the epidemiological characteristics, risk factors associated with TB, and drug resistance to TB in luanda
8	Fortunato; Sant’Anna, 2011 [24]	International journal of tuberculosis and lung disease	To investigate the use of the brazilian Ministry of Health’s guidelines for the simultaneous use of chest X-rays and the Mantoux tuberculin skin test to identify children with latent tuberculosis infection (LTBI); and to follow up on contacts for 6 months
9	Robbiati et al., 2022 [29]	Frontiers in public health	Describe the process of developing an electronic medical record system and share the lessons learned
10	Brady; Vita, 2018 [5]	Journal of public health	Ask healthcare professionals involved in tuberculosis control what they consider to be the drivers of the tuberculosis epidemic in Angola
11	Dos santos et al., 2019 [28]	Archivos de bronconeumologia	To evaluate the effect of incentives (basic food basket) on treatment outcomes for patients with tuberculosis at the tuberculosis clinic in huambo
12	Nindia et al., 2025 [23]	Open forum Infectious diseases	To implement systematic screening for TB using molecular tests on stool samples from children admitted to the acute Malnutrition unit (UMA) at a rural hospital in benguela province, Angola
13	Vita et al., 2024 [6]	Tropical medicine and Infectious disease	To identify and analyze the risk factors associated with loss to follow-up in TB treatment at the luanda sanatorium hospital

**TABLE 2:** Characteristics of studies included in the scoping review on comprehensive TB care in Angola (Angola, 2025).

No.	Author/Year of publication	Data analysis	Actions taken	Challenges and opportunities in implementing or carrying out the actions
1	Martínez-Camprecios et al., 2024 [20]	Descriptive	- Contact tracing for index cases of pulmonary TB, followed by a clinical examination; - Availability of smear microscopy and rapid molecular testing; - Availability of chest X-rays; - Availability of HIV testing; - Availability of preventive treatment for tuberculosis; - Availability of treatment for contacts with active TB	Challenges: - Low adherence among contacts to the screening process, due to socioeconomic barriers; - Low sputum yield in children; - The COVID-19 pandemic; - Lack of free testing during certain periods; - Difficulties in reaching pediatric contacts; - Insufficient counseling for contacts and family members; - Shortage of medications; - Lack of knowledge among healthcare professionals regarding other drugs for preventive treatment besides isoniazid; Strengths: - Involvement of community health workers (CHWs) in community-based DOT - Contact tracing in the community - Free testing, clinical evaluation, and collection of gastric lavage samples from children - Equipes médicas extra - Abertura do serviço um dia a mais
2	Faust et al., 2020 [21]	Descriptive	- Preventive treatment of tuberculosis with isoniazid; - X-rays for contact tracing	Challenges: - Lack of guidelines for the treatment of latent tuberculosis infection (LTBI); - Prioritization of active TB treatment and contact tracing among people living with HIV; - Financial barriers to implementing the program and IGRA testing; - Lack of screening for latent TB infection in children and other at-risk populations; - Failure to use the PPD test for screening latent TB infection; - Inadequate laboratory infrastructure and logistics, and a shortage of human resources
3	Oliveira; Martinez; Rocha, 2014 [18]	Generalized linear models (log-binomial model)	−90% of the children had received the BCG vaccine	- Did not mention
4	Umar; Kabamba, 2016 [19]	Not applicable	- Follow-up visits to identify individuals who have missed their vaccinations; - Checking for BCG vaccination scars	Challenges: - The need to travel to another country (Namibia) to access more affordable maternal and child health services; - Lack of awareness that vaccinations and tuberculosis treatment are free; - Lack of involvement by community leaders in policy decisions aimed at improving the healthcare system and service delivery
5	Doveren, 2001 [25]	Not applicable	- Followed the national guidelines of the tuberculosis program; - Health education; - Tracking down patients who have stopped treatment; - Implementation of DOTS	- Missing test results for people being treated for TB; - Tests conducted in private laboratories; - Lack of supplies needed to perform tests; - Drug shortages; - Lack of information on treatment outcomes; - High rate of loss to follow-up; - War conditions Strengths: - Optimism among the team and people affected by TB; - Provision of fruits and fish to improve nutritional status; - Community health agents (ACS) continued to monitor cases
6	Cameia, 2020 [26]	Content analysis	- Case tracking; - Provision of diagnostic resources in specialized and non-specialized care settings; - Availability of tests; - Multidisciplinary care; - Provision of medications and incentives; - Counseling	Challenges: - Misconceptions about TB/HIV coinfection affecting treatment adherence - Stigma and discrimination; - Lack of supplies; - Lack of an action plan for people who discontinue treatment;
7	Sebastião et al., 2022 [22]	Absolute and relative frequencies, the chi-square test, and logistic regression	- MTB identification test; - Antibiotic susceptibility test	Challenges: - Lack of an epidemiological surveillance system; - Underreporting of cases
8	Fortunato; Sant’Anna, 2011 [23]	Descriptive	- Chest X-rays and tuberculin skin tests; - Clinical evaluation; - Nutritional status assessment; - HIV serology; - Treatment of children with active tuberculosis	Challenges: - Lack of systematic contact tracing; - Low national income
9	Robbiati et al., 2022 [28]	Not applicable	- Development of an electronic medical record system for case documentation	Challenges: - High costs associated with purchasing and maintaining the electronic medical record system; - Poor implementation planning; - Problems with electricity supply and internet connectivity; - Limited computer skills among users
10	Brady; Vita, 2018 [29]	Interpretive phenomenological analysis	- They do not mention specific actions—they only discuss the challenges of controlling TB	Challenges: - Interruption of treatment; - Lack of resources in rural areas; - Failure by healthcare professionals to comply with biosafety measures; - Lack of healthcare professionals trained in new strategies for controlling and preventing tuberculosis
11	Dos santos et al., 2019 [27]	Descriptive statistics and the Mann-whitney U test	- Distribution of food baskets and food to people with TB who are undergoing treatment	Challenges: - Lack of resources - Interruptions in the provision of food baskets to people undergoing treatment
12	Nindia et al., 2025 [22]	Descriptive statistics, chi-square test or Fisher’s exact test, and Student’s t-test or Mann-whitney test	- Systematic TB screening using Xpert MTB/RIF Ultra; - Clinical evaluation; - Immediate treatment for TB; - Rapid HIV testing	Challenges: - A significant number of invalid results Facilitating factors: - High acceptance of the strategy, - Simplicity of stool collection
13	Vita et al., 2024 [6]	Descriptive statistics, chi-square test or Fisher’s exact test, Student’s t-test or Mann-whitney test, and multivariate logistic regression analysis	- Clinical evaluation; - Smear test; - Xpert MTB/RI molecular test; - Chest X-ray; - Treatment	Challenges: - Cases treated without microbiological confirmation; - Inability to track whether patients who did not return for follow-up were treated at another healthcare facility; - Lack of computerization in healthcare facilities; - Food insecurity, including among hospitalized patients Strengths: - Free access to medications

Five themes emerged from the thematic analysis. The first theme highlighted the role of prevention and diagnostic capacity as essential components of comprehensive TB care in Angola. This category included interventions such as BCG vaccination and monitoring of its implementation [19, 20]; contact tracing [21, 22]; systematic screening of children and other vulnerable groups [21, 22, 23]; the use of combined diagnostic approaches, including clinical assessment, tuberculin skin testing, chest radiography, sputum smear microscopy, and molecular rapid tests [6, 21, 23, 24]; and strengthening of diagnostic capacity through drug susceptibility testing and HIV testing [21, 23, 24, 25]. However, the implementation of these strategies was challenged by low adherence to contact tracing activities [21] and insufficient coverage of diagnostic testing [26].

The second theme, “Continuity of care and treatment support as dimensions of comprehensive care,” highlighted that, despite advances in access to diagnosis and treatment initiation, maintaining continuity of care remains a major challenge for TB control in Angola. The findings indicate that treatment adherence and retention in care depend not only on the availability of medicines but also on sustained support throughout the treatment pathway. Strategies identified across the included studies included the implementation of DOTS [26] and community-based follow-up [21]; active retrieval of patients lost to follow-up [20, 26] and follow-up visits [20, 21]; counselling and multidisciplinary care [27]; provision of incentives, such as food baskets and nutritional support [27, 28]; and free access of medicines [6, 27].

Despite these efforts, the included studies identified persistent barriers affecting treatment retention and continuity of care. These barriers involved both health system limitations and social determinants, including the absence of structured strategies for retrieving patients lost to follow-up [27]; limited digital literacy among healthcare professionals for the use of electronic medical records [29]; stigma and discrimination [27]; and food insecurity and discontinuation of social support interventions [27, 28].

The third theme, “Structural and organizational barriers to comprehensive TB care”, highlighted how health system limitations constrain the implementation and sustainability of TB control strategies in Angola. The identified barriers reflected interconnected challenges involving supply chains, service organization, health information systems, workforce capacity, and surveillance structures. These included drug stockouts [21, 26]; insufficient diagnostic supplies and materials [26, 27]; the absence of guidelines for the diagnosis and management of latent tuberculosis infection (LTBI) [22]; inadequate laboratory infrastructure, insufficient human resources, and limited professional training [5, 22].

Additionally, the lack of electronic medical records, combined with electricity and internet connectivity constraints, hindered the implementation of health information systems [29]. Gaps in epidemiological surveillance, underreporting of cases [25], limited availability of treatment outcome data [26]; and treatment initiation without bacteriological confirmation [6] further demonstrated weaknesses in the capacity of the health system to ensure comprehensive TB care.

The fourth theme, “Addressing social determinants of health to achieve comprehensive TB care”, highlighted that TB control efforts in Angola occur within a context of social vulnerability, health inequities, territorial disparities, and periods of crisis that influence access to and continuity of care. The findings indicate that factors beyond healthcare delivery contribute to TB-related vulnerabilities and may limit the effectiveness of comprehensive care strategies. Identified challenges included limited awareness of the availability of free health services [20]; stigma and discrimination [27]; food insecurity [6]; armed conflicts [26]; and the COVID-19 pandemic [21].

The last theme, “Community-based strategies, access facilitation, social support, and health information sharing as drivers of comprehensive TB care”, highlighted existing programmatic and operational strengths that support TB care delivery in Angola. The findings demonstrated the contribution of community-based approaches and patient support mechanisms to improving access, follow-up, and continuity of care.

Key strategies included the involvement of community health workers (CHWs) in case follow-up [21, 26], community-based contact tracing [21], free access to diagnostic tests [6, 21], clinical evaluation, and medicines [23, 24, 27], the use of gastric lavage samples to improve TB diagnosis among children [21, 23], expansion of medical teams and service availability [21, 26], multidisciplinary care and counselling [27], nutritional and food support [26, 28], health worker commitment and engagement [26] and implementation of electronic medical records [29]. However, despite these advances, greater involvement of community leaders in policy-making processes remains an important area for strengthening comprehensive TB care [20].

## Discussion

This scoping review mapped and synthesized the available evidence on comprehensive TB care in Angola, providing an integrated overview of the strategies implemented, as well as the barriers and opportunities influencing TB care delivery. The findings indicate that, despite important advances in TB surveillance and care over the past two decades, persistent structural, organizational, and social challenges continue to compromise the delivery of comprehensive, patient-centred care and limit progress toward the global TB control targets.

Regarding TB prevention, two studies reported the use of BCG vaccination in Angola [19, 20] reinforcing its continued importance in preventing severe forms of TB during early childhood. One study [20], highlighted that, despite the vaccine being provided free of charge, barriers to access remain, underscoring the need for public awareness initiatives and organizational improvements to expand vaccination coverage.

Other preventive actions implemented in the Angolan context include the use of the tuberculin skin test (TST) [24] to identify TB infection and isoniazid preventive treatment [21, 25, 26]. These findings suggest limited implementation of current recommendations for the diagnosis and treatment of latent TB infection (LTBI), as interferon-gamma release assays (IGRA) are now available and offer greater specificity than the TST, alongside shorter preventive treatment regimens such as 3HP, 3RH, and 4R [30, 31].

Considering that treatment TB infection is widely recognized as a key public health strategy to prevent new MTB infections and progression to active disease [21]. Therefore, Angola should establish or update national guidelines and strengthen the capacity of health professionals to implement LTBI diagnosis and treatment. In addition, efforts should focus on expanding screening infection among contacts of index cases, people living with HIV, and individuals with other immunosuppressive conditionsHealth education and counseling interventions are also needed to encourage contact participation in screening and treatment while reducing stigma, discrimination, and misconceptions related to TB the disease and its comorbidities [27].

The expansion of TB diagnostic capacity in Angola reflects important progress toward comprehensive TB care by promoting early case detection and timely diagnosis. Studies included in this review reported the implementation of clinical evaluation, chest radiography, sputum smear microscopy, rapid molecular testing (GeneXpert MTB/RIF), culture, drug susceptibility testing, and assessment for coinfections [6, 21, 23, 25, 27]. Together, these strategies contribute to strengthening TB control in line with the End TB Strategy.

Despite these advances, important implementation barriers remain. The included studies reported interruptions in the availability of free diagnostic testing [21], shortages of diagnostic supplies, difficulties in communicating or obtaining test results, particularly from private services, and diagnostic challenges in specific populations, such as children, who may require procedures such as gastric lavage [21, 27, 29]. These findings indicate that expanding diagnostic technologies alone is insufficient without strengthening laboratory capacity, service organization, and equitable access to diagnostic services.

Pharmacological treatment for TB is provided free within the Angolan health system [21, 23, 26, 27]. However, interruptions in treatment continuity remain frequent [21, 26], contributing to high rates of loss to follow-up. This challenge is compounded by difficulties actively tracing patients who miss scheduled appointments, and by the absence of computerized systems to support treatment monitoring. In addition, structural barriers affecting access, continuity, and coordination of care, together with social vulnerabilities, food insecurity, stigma, and discrimination, further compromise treatment adherence and the delivery of comprehensive TB care.

Recent WHO guidelines emphasize that the goals of the End TB Strategy can only be achieved when TB diagnosis, treatment, and prevention are integrated within the context of universal health coverage [8]. Angola has not yet fully achieved this standard, highlighting the need to expand and strengthen TB services through adequately equipped health facilities, reliable supply chains, and improved access to care across municipalities, communities, and rural areas.

Angola reports high proportions of unknown treatment outcomes and loss to follow-up [26], highlighting weaknesses in treatment monitoring and continuity of care. Strengthening active patient tracing, DOT implementation, and mechanisms for information sharing across health services are essential to improve follow-up, particularly through the use of electronic health records. However, implementation of these systems remains challenging due to high costs, unreliable electricity and internet connectivity, limited digital literacy, insufficient computerization of health facilities [29, 32].

A study conducted in Brazil [33] demonstrated that patient medical records are essential tools for information sharing among healthcare professionals and for coordinating TB care. Therefore, the absence of electronic health records in Angola may limit the communication between services, weaken care coordination, and hinder the delivery of comprehensive TB care. The implementation DOT strategy differs across Angolan provinces, available evidence suggests that this approach may not have been fully implemented nationwide, potentially contributing to the high rates of loss to follow-up previously described. Evidence from southwestern Iran demonstrated that DOTS can contribute to substantial improvements in the TB situation of low-income settings through a cost-effective and sustainable approach, reducing mortality, prevalence, incidence of new cases, and drug resistance [34]. However, DOT implementation should be adapted to local contexts and health system capacities. Different models, including home-based strategies supported by community health workers [26], facility-based approaches, and digitally supported follow-up, may be considered according to available resources and operational feasibility.

A study conducted in Índia highlighted that TB is not solely a biomedical and public health issue, but also a socially determined disease. The persistence of poverty and social inequalities continues to influence TB vulnerability and outcomes. Therefore, effective TB control requires multiprofessional and intersectoral interventions alongside strengthened public policies addressing food insecurity, poverty, and educational inequalities [35]. These findings are particularly relevant for Angola, where social vulnerabilities and structural barriers remain important challenges for achieving comprehensive TB care.

In this context, comprehensive care requires addressing the socioeconomic, geographic, and cultural factors that influence the health–disease process, as well as developing responses that extend beyond the traditional boundaries of healthcare services. TB interventions are more likely to be effective when strategies and policies are adapted to the local context and respond to existing territorial and social inequalities. This perspective is particularly relevant in Angola where challenges such as stigma, food insecurity, and limited health literacy require intersectoral approaches rather than healthcare-based interventions alone.

Community-based interventions identified in the included studies contribute to improving treatment adherence and mitigating social and economic factors that exacerbate the TB epidemic [27, 35]. Strengthening TB control efforts requires coordinated actions involving health services, social assistance, education, and food security, as well as policies that address prevention, diagnosis, treatment, and psychosocial support. Empowering community actors through advocacy and social mobilization is essential to increase awareness promote shared responsibility, and strengthen social participation in TB control. Effective responses at the territorial level dependon coordinated action and collaboration among multiple stakeholders.

The identified strengths indicate actionable opportunities to enhance comprehensive TB care in Angola. A common characteristic of these strengths is their potential to bring health systems closer to the realities experienced by people with TB through community-based approaches, improved access to diagnosis, multidisciplinary care, and responses tailored to patients’ needs. These findings suggest that strengthening TB programmes does not necessarily depend on advanced technological solutions, but rather on feasible, context-adapted, and sustainable organizational strategies capable of improving care delivery within local health systems.

One of the studies included in this review, conducted in Angola and other countries [22], highlighted differences in LTBI management across settings. Compared with Brazil, Lesotho, Mozambique, Russia, and Zambia, Angola lacked national guidelines for LTBI management and remained focused mainly on the treatment of active TB disease. Similar to Kenya and the Democratic Republic of the Congo, financial barriers limited the implementation of LTBI strategies. Additionally, TST and IGRAs were not incorporated into routine practice within the National TB Control Programme, with TST available only in selected provinces. These findings indicate that expanding TB preventive treatment remains an important challenge for Angola and requires alignment with global recommendations to reduce progression to active TB and achieve End TB Strategy targets.

In relation to the treatment and follow-up of people with active TB, no comparative studies involving Angola and other countries were identified in this review, highlighting an important gap in the scientific literature. Therefore, a comprehensive understanding of TB epidemiology in Angola, considering its political, social, and health system context, is essential to inform the adaptation and strengthening of effective TB control strategies. These strategies should integrate prevention, diagnosis, treatment, and continuity of care to contribute to achieving the Sustainable Development Goals and the WHO targets for *End TB* as a public health problem [2].

Importantly, the TB response in Angola depends not only on the availability of technically recommended interventions, but also on the health system’s capacity to ensure their continuity, accessibility, local adaptation, and sustainability. By synthesizing the available evidence, this review addresses an empirical gap by providing the first comprehensive overview of TB care actions in Angola, a system-level gap by linking surveillance, health system capacity, and care delivery, and a contextual gap by generating context-specific evidence from a high-burden and resource-constrained setting.

This study has several limitations. First, despite the structured search strategy based on controlled vocabulary and free-text terms, the possibility of missing relevant studies cannot be excluded, which may have limited the breadth of the evidence identified. Second, although study selection was independently conducted by reviewers, inter-rater agreement was not assessed using Cohen’s kappa statistic, which may have introduced some degree of subjectivity into the selection process. Finally, in accordance with the scoping review methodology and considering the descriptive nature of the synthesis and the methodological heterogeneity among the 13 included studies, methodological quality appraisal was not performed. Therefore, the strength and certainty of the available evidence should be interpreted with caution.

Additionally, publication bias may have influenced the evidence identified, as studies with negative or inconclusive findings are less likely to be published. Regarding grey literature, the search was conducted exclusively through Google Scholar; therefore, the absence of additional sources, such as government reports, technical documents, and unpublished data, may have limited the identification of ongoing interventions and undocumented challenges within the TB response. Finally, the exclusive focus on Angola limits the transferability of findings to other African countries with different epidemiological and health system contexts.

Based on the findings of this review, future TB control efforts in Angola should prioritize the expansion and decentralization of TB services, particularly in underserved rural areas. The integration of digital health technologies, improved access to rapid diagnostic tools, and strengthened community-based strategies may contribute to improving treatment adherence, early diagnosis, and continuity of care. These actions should be accompanied by investments in health professional training, community health workers, reliable supply chains, and intersectoral policies addressing social determinants of TB. Generating locally relevant evidence through operational research will also be essential to guide sustainable and context-adapted models of comprehensive TB care.

In conclusion, this study highlights important advances in important advances in comprehensive TB care in Angola over the past two decades. However, persistent structural, organizational, and socioeconomic challenges continue to limit progress, including gaps in contact tracing, interruptions in diagnostic testing and drug supply, and loss to follow-up. Strengthening TB control requires integrated policies focused on health workforce training, expanded access to rapid diagnostic tools, decentralized service delivery, community engagement, and robust surveillance systems. Community-based strategies, including DOT and active case finding, represent important opportunities to improve access, reduce barriers, and enhance continuity of care, particularly in underserved areas. These actions are essential to support Angola’s progress toward the WHO End TB Strategy targets and contribute to ending TB as a public health problem.
