# Supplementary material for: Strengthening comprehensive tuberculosis care in Angola: a scoping review of strategies, challenges, and potentialities in a high-burden setting
Source: Public Health Rev. 2026 Aug 27;47:1609466. doi: 10.3389/phrs.2026.1609466 (PMC13558124; doi:10.3389/phrs.2026.1609466)
Supplement: Supplementary file 1 [file Table1.docx]

**Supplementary Material**

**Table 1.** Literature search strategies for the scoping review on comprehensive tuberculosis care in Angola (Angola, 2025).

| **Databases** | **Search strategy** |
| --- | --- |
| **PUBMED / MEDLINE** | ("tuberculosis"[MeSH Terms] OR "tuberculosis"[All Fields] OR "tb"[All Fields] OR "mdr-tb"[All Fields] OR "tb-hiv"[All Fields]) AND ("prevent"[All Fields] OR "preventability"[All Fields] OR "preventable"[All Fields] OR "preventative"[All Fields] OR "preventatively"[All Fields] OR "preventatives"[All Fields] OR "prevented"[All Fields] OR "preventing"[All Fields] OR "prevention and control"[MeSH Subheading] OR "prevention and control"[All Fields] OR "prevention"[All Fields] OR "preventive"[All Fields] OR "preventively"[All Fields] OR "preventives"[All Fields] OR "prevents"[All Fields] OR "preventive actions"[All Fields] OR "diagnosis"[MeSH Subheading] OR "diagnosis"[All Fields] OR "screening"[All Fields] OR "mass screening"[MeSH Terms] OR "mass screening"[All Fields] OR "screen"[All Fields] OR "screenings"[All Fields] OR "screened"[All Fields] OR "screens"[All Fields] OR "diagnosable"[All Fields] "diagnosis"[MeSH Terms] OR "diagnose"[All Fields] OR "diagnosed"[All Fields] OR "diagnoses"[All Fields] OR "diagnosing"[All Fields] OR "diagnostic"[All Fields] OR "diagnostical"[All Fields] OR "diagnostically"[All Fields] OR "diagnostics"[All Fields] OR "care"[All Fields] OR "therapeutical"[All Fields] OR "therapeutically"[All Fields] OR "therapeuticals"[All Fields] OR "therapeutics"[MeSH Terms] OR "therapeutics"[All Fields] OR "therapeutic"[All Fields] OR "therapies"[All Fields] OR "therapy"[MeSH Subheading] OR "therapy"[All Fields] OR "monitor"[All Fields] OR "monitorable"[All Fields] OR "monitored"[All Fields] OR "monitoring"[All Fields] OR "monitorings"[All Fields] OR "monitorization"[All Fields] OR "monitorize"[All Fields] OR "monitorized"[All Fields] OR "monitors"[All Fields] OR "follow-up"[All Fields] OR "directly observed treatment"[All Fields] OR "surveillance"[All Fields] OR "surveilance"[All Fields] OR "surveillances"[All Fields] OR "surveilled"[All Fields] OR "surveillence"[All Fields]) AND ("angola"[MeSH Terms] OR "angola"[All Fields] OR "angolan"[All Fields] OR "angolans"[All Fields]) |
| **EMBASE** | #1 'tuberculosis'/exp OR tuberculosis OR 'tb'/exp OR tb OR 'mdr-tb'/exp OR 'mdr-tb' OR 'tb-hiv'  #2 ('prevention'/exp OR prevention OR 'preventive actions' OR 'screening'/exp OR screening OR 'diagnosis'/exp OR diagnosis OR 'diagnostic'/exp OR diagnostic OR 'care'/exp OR care OR 'control'/exp OR control OR therapeutic OR 'therapy'/exp OR therapy OR 'monitoring'/exp OR monitoring OR 'follow-up'/exp OR 'follow-up' OR 'directly observed treatment' OR 'surveillance'/exp OR surveillance) AND [embase]/lim  #3 ('angola'/exp OR angola OR 'angolan'/exp OR angolan OR 'angolans'/exp OR angolans) AND [embase]/lim  #4 #1 AND #2 AND #3 |
| **LILACS*** | tuberculosis AND angola AND db:("LILACS") AND instance:"lilacsplus" |
| **Web of Science** | tuberculosis OR tb OR "mdr-tb" OR "tb-hiv" (Topic) and prevention OR "preventive actions " OR screening OR diagnosis OR diagnostic OR care OR control OR therapeutic OR therapy OR monitoring OR "follow-up" OR "directly observed treatment" OR surveillance (Topic) and Angola OR Angolan OR Angolans (Topic) |
| **SCOPUS** | TITLE-ABS-KEY (tuberculosis OR tb OR "mdr-tb" OR "tb-hiv") AND TITLE-ABS-KEY (prevention OR "preventive actions " OR screening OR diagnosis OR diagnostic OR care OR control OR therapeutic OR therapy OR monitoring OR "follow-up" OR "directly observed treatment" OR surveillance) AND TITLE-ABS-KEY (Angola OR Angolan OR Angolans) |
| **GOOGLE SCHOLAR** | tuberculosis surveillance angola  tuberculose vigilância angola |

* No studies were identified in the LILACS database.
 Source: Adapted from Page et al. (2020)
